# State-of-the-Art of Non-Radiative, Non-Visual Spine Sensing with a Focus on Sensing Forces, Vibrations and Bioelectrical Properties: A Systematic Review

**DOI:** 10.3390/s23198094

**Published:** 2023-09-26

**Authors:** Maikel Timmermans, Aidana Massalimova, Ruixuan Li, Ayoob Davoodi, Quentin Goossens, Kenan Niu, Emmanuel Vander Poorten, Philipp Fürnstahl, Kathleen Denis

**Affiliations:** 1 KU Leuven, Department of Mechanical Engineering, BioMechanics (BMe), Smart Instrumentation, 3000 Leuven, Belgium; goossens.quentin@gmail.com (Q.G.); kathleen.denis@kuleuven.be (K.D.); 2 Research in Orthopedic Computer Science (ROCS), University Hospital Balgrist, University of Zurich, 8008 Zurich, Switzerland; aidana.massalimova@balgrist.ch (A.M.); philipp.fuernstahl@balgrist.ch (P.F.); 3 KU Leuven, Department of Mechanical Engineering, Robot-Assisted Surgery Group (RAS), 3000 Leuven, Belgium; ruixuan.li@kuleuven.be (R.L.); ayoob.davoodi@kuleuven.be (A.D.); k.niu@utwente.nl (K.N.); emmanuel.vanderpoorten@kuleuven.be (E.V.P.)

**Keywords:** spine surgery, sensor, robotic, force, vibration, bioelectrical, systematic review

## Abstract

In the research field of robotic spine surgery, there is a big upcoming momentum for surgeon-like autonomous behaviour and surgical accuracy in robotics which goes beyond the standard engineering notions such as geometric precision. The objective of this review is to present an overview of the state of the art in non-visual, non-radiative spine sensing for the enhancement of surgical techniques in robotic automation. It provides a vantage point that facilitates experimentation and guides new research projects to what has not been investigated or integrated in surgical robotics. Studies were identified, selected and processed according to the PRISMA guidelines. Relevant study characteristics that were searched for include the sensor type and measured feature, the surgical action, the tested sample, the method for data analysis and the system’s accuracy of state identification. The 6DOF f/t sensor, the microphone and the electromyography probe were the most commonly used sensors in each category, respectively. The performance of the electromyography probe is unsatisfactory in terms of preventing nerve damage as it can only signal after the nerve is disturbed. Feature thresholding and artificial neural networks were the most common decision algorithms for state identification. The fusion of different sensor data in the decision algorithm improved the accuracy of state identification.

## 1. Introduction

Spine surgery is a complex procedure, the outcome of which often depends on the experience of the surgeon. Pedicle screw placement has become one of the most practiced procedures in spine surgery but screw misplacement remains a major complication, even for very experienced surgeons, as pedicle screw misplacement rates have been reported between 5 and 41% in the lumbar spine and between 3 and 55% in the thoracic spine [1]. A review study by Tsai et al. investigating the revision spinal surgery in 10,350 patients found that the most common surgical cause of reoperation within a week was screw malpositioning followed by inadequate decompression and epidural hematoma [2]. Long-term causes of reoperation after spine surgery were listed by Dymén et al. with the infection as a leading cause followed by implant-related causes (implant migration, implant breakage, misplaced implant and pain over the implant site) and haemorrhage/hematoma [3]. Not only do these issues cause revision surgery with high complexity, but they can also lead to serious postoperative complications, severe therapeutic consequences, and potential neurologic decline [4].

A major cause of the mentioned issues is the limited visualisation in the surgical site and limited feedback to the surgeon of where the spinal implant is within the anatomy during the insertion and which tissue is being manipulated by surgical instruments such as a drill, probe, or forceps. In those cases, tactile and auditory senses are the only available feedback to the surgeon. The problem of limited visualisation and feedback becomes more evident in the case of minimally invasive spine surgery (MISS). A review by Camacho et al. on MISS in spinal trauma lists that MISS has many advantages over open surgery such as reduced blood loss, the lower risk of surgical site infection and an improvement in postoperative care and rehabilitation [5]. However, visualisation is even more difficult in that case and must often rely on radiative imaging techniques. Less invasive spine surgery has been shown to have increased radiation exposure to the surgeon and patient [6]. Furthermore, a study by Timur M. Murakov showed that protective equipment such as a lead gown can still let 80% of the radiation reach the surgeon’s body when they remain within 2 ft of the emitter, which is the typical distance within which the surgeon and assistants operate [7]. Radiation exposure to the patient and medical staff should be minimised as it leads to serious health risks and carcinogenic effects through cellular damage and DNA lesion [8].

The technology in the operating theatre has evolved together with the problems that are being faced and many inventions have been made to improve intraoperative visualisation for (minimally invasive) spine surgery. The latest advancements include augmented reality wearables to project the underlying anatomical structures and pre-planned operating paths on the patient to improve the operative workflow [9]. However, only the surgeon’s visual sense is aided through this technology, while tactile and auditory feedback are also hindered in MISS. Several review studies have shown that, for the proper intraoperative tissue classification, it is essential to use a combination of sensing techniques from different modalities to mimic our human ability to combine our different senses in a decision-making process and to accommodate for the much-needed sensor redundancy in complex surgical tasks [10,11]. According to Qu et al. the future direction for spine research should focus on information processing through multisensor technology to ensure effectiveness and safety as tissue recognition is affected by differences in surgical instruments, operating methods, surgical paths, operating speeds, and other factors [10]. Applying a wide variety of sensors and sensing techniques can potentially increase the robustness of detection and classification algorithms. The current surge in artificial intelligence-centred investigations is beneficial for the goal of elaborate multisensory technology for accuracy improvement in spine surgery, as enhanced pattern recognition and data fusion are now available through established machine learning models [12,13].

Such human-like sensing and decision making could possibly be performed and even enhanced by an integrated surgical robot system, as robots are currently employed more and more in the operating theatre because of their high precision and repeatability. In the research field of robotic spine surgery, there is a big upcoming momentum for surgeon-like autonomous behaviour and surgical accuracy in robotics which goes beyond the standard engineering notions such as geometric precision or resolution. As Staub et al. concludes that current technology does not allow for true autonomous robotic spine surgery but rather for the guidance of the surgeon, it shows that now is the time to boost that research [14]. A systematic review study by Ghasem et al. compared the outcome of robotic and free-hand spine surgery and found that there was an improved accuracy of screw placement with robotic surgery but that preplanned drilling pathways were shown to be altered intraoperative environmental factors, such as soft tissue pressure applied to the drill or skiving off bony surfaces [15]. It is exemplary of how high precision does not guarantee safety and that the integration of multisensor technology and information processing is necessary to establish robotic spine surgery in the operating theatre. The same issues that human surgeons face with visualisation are also still present in robotic surgery and a review study from D’Souza et al. found no conclusive differences between robotic and free-hand surgery [16].

To summarise, surgeons and surgical robotic systems are facing limited sensory feedback in spine surgery which can lead to severe complications such as pedicle screw malpositioning and hematoma by unplanned breach during spinal drilling. Moreover, standard visualisation and imaging techniques in the operating theatre cause radiation exposure to the patient and medical staff, which should be minimised due to its health hazards. This systematic review investigates which sensor types and sensing methods can be applied in spine surgery and integrated in a multisensor platform to provide redundancy in the operational feedback to surgeon or robot. As the visual field is limited in spine surgery and there is a shift towards MISS, this study focuses on non-visual, non-radiative technology. The investigated categories of sensing methods were based on two review studies on tissue classification methods in spine surgery [10,11]. The three categories include the sensing of force and torque, sound and vibration, and bioelectrical properties. The objectives of this review are to provide an overview of the state-of-the-art in sensing technologies in spine surgery, to identify trends in the use of certain sensors or in data processing and to investigate whether there are research gaps for future developments in the field. The following research questions were introduced in order to systemise the search for adequate sensors.

- Which sensors are the state-of-the-art in non-visual, non-radiative, intraoperative sensing for spinal surgery with a focus on sensing forces, vibrations and bioelectrical properties?
- Which physical properties are measured?
- How are the gathered data processed?
- What is the accuracy of the proposed measurement system?

This systematic review is novel in the fact that it tries to offer a condensed summary of the available and tested sensor technologies for future research in robotic spine surgery. It provides a vantage point that facilitates experiment and prototype design and guides new research projects to what has not been thoroughly investigated or integrated in robotics to date. The included tables listing the sensors, their application and their performance can be used to select which sensors or algorithms for signal processing can be combined to perform real-time decision-making based on a multitude of feedback signals.

**Figure 1:**
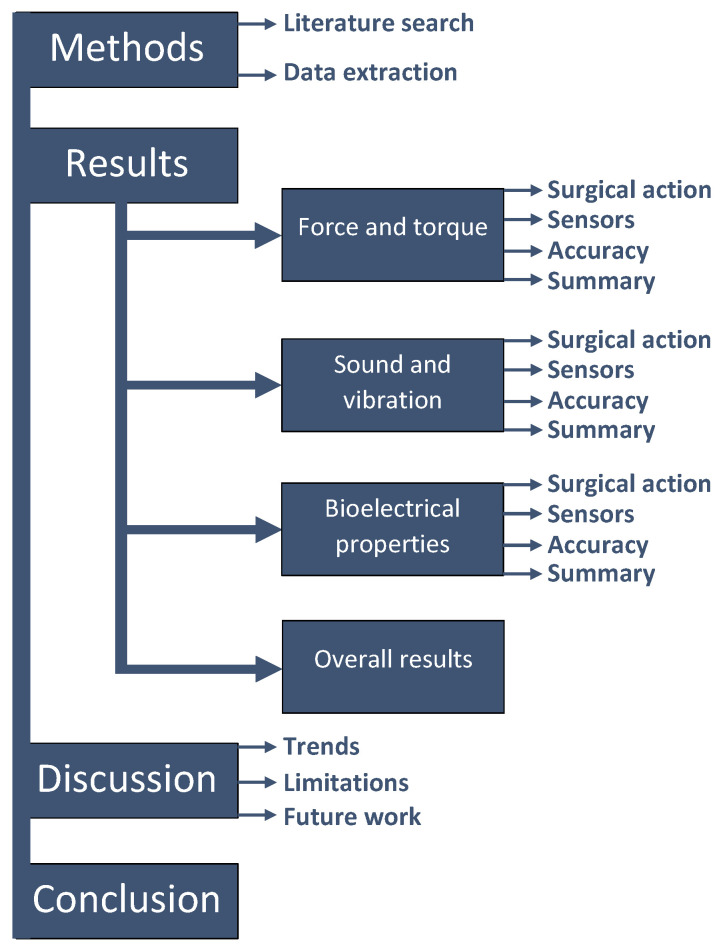
Structure of this systematic review. The methods section describes how the literature review was performed. In the results section, the literature is divided into three categories based on what type of feedback signal the sensor measures. Connections between the literature are elaborated on in the discussion section.

The structure of this systematic review is shown in Figure 1. The methods section describes how the literature search was performed, as well as the applied inclusion criteria and which data were extracted from the included studies. The results section covers the three categories of sensing. For each category, the surgical action that is performed in each study, the sensor type, the information processing method, the purpose and the accuracy of the sensing method is discussed and shortly summarised. The discussion section covers the development trends that emerge from the literature review, the limitations of this study and suggestions for future work. Finally, the conclusions section formulates an answer to the research questions of this systematic review.

## 2. Methods

### 2.1. Literature Search and Inclusion Criteria

Preferred Reporting Items for Systematic Reviews and Meta-Analysis (PRISMA) guidelines [17] were followed to perform this systematic review on the state-of-the-art in non-radiative and non-visual spine sensing. A search in four databases (PubMed, IEEE Xplore, Ovid and Scopus) was performed with following search terms: spine, vertebra, pedicle, intervertebral disc, screw, instrumentation, fusion, discectomy, drill*, mill*, cutting, condition, state, tissue, breach, perforat*, breakthrough, sensor, distinguish*, classif*, discriminat* recogni*, identif*, force, torque, haptic, sound, acoustic, vibration, emission impedance, resistance, conductivity, electrical. No further search filters were applied in the aforementioned databases. The keywords were combined in a string that was used as a search input in the databases. An example is given for the one used in PubMed: (condition OR state OR tissue OR breach OR perforat* OR breakthrough) AND (screw OR instrumentation OR fusion OR discectomy OR drill* OR mill* OR cutting) AND (sensor OR distinguish* OR classif* OR discriminat* OR recogni* OR identif* OR quantif*) AND (spine OR vertebra OR pedicle OR intervertebral disc) AND (force OR torque OR haptic OR sound OR acoustic OR vibration OR emission OR impedance OR resistance OR conductiv* OR electric*). The strings for the other databases were similar, with the exception that some databases accept no or a limited number of wildcards. Then, all the search terms with wildcards were replaced by their noun. e.g., recogni* was replaced by recognition. The search was performed within the title, abstract and keywords of the studies in each database. Two independent reviewers used the exact same strings of keywords to search the four databases, completely blinded to each other’s results to reduce the risk of bias. The search was performed on 26 April 2021 by the first reviewer and on 7 May 2021 by the second one. An iteration of the search was performed on 17 August 2022. The resulting studies were reviewed based on title and abstract. The following inclusion criteria based on relevance to the topic were utilised:

1. Sensing is performed on bone tissue or artificial (but mechanically similar) alternatives.
2. The sensors fall within the category of measuring force and torque, sound and vibration or bioelectrical properties.
3. The proposed sensing method is non-radiative and non-visual.

The list of selected studies of each reviewer was compared and disagreements (*n* = 5, three studies included, two excluded) were resolved by consensus. Consecutively, the review was performed on the full text for all studies that were agreed on. The following eligibility criteria were acquired:

1. The studies are written in English and a full text is available.
2. The journal in which the study was published has a SJR score with a Q-index of Q1 or Q2.
3. The studies are published not earlier than 2005.

**Figure 2:**
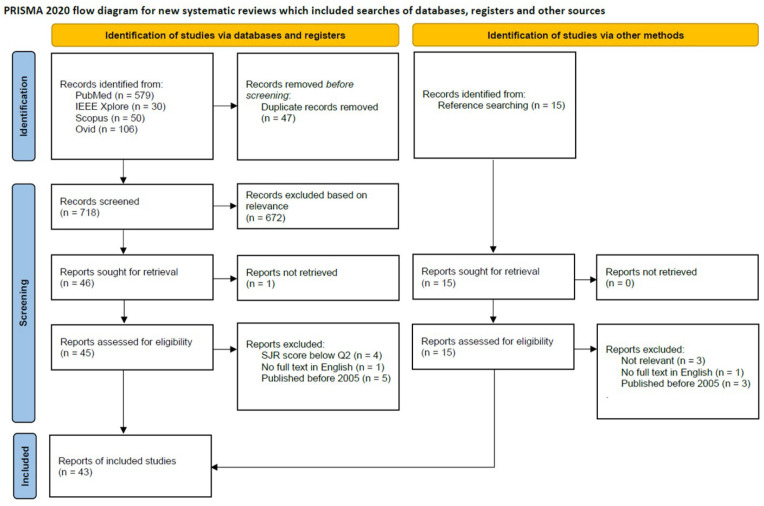
PRISMA flow diagram of this systematic review. It gives a schematic overview of where the literature was found and how the final number of included studies was obtained through the elimination of studies that do not meet the inclusion and eligibility criteria.

The studies that did not meet all inclusion criteria and all eligibility criteria were excluded from this systematic review. No further exclusion criteria were applied. Beyond the systematic search via databases, fifteen studies were identified through the analysis of the references in the studies that came out of the systematic search and that met the above-mentioned inclusion and eligibility criteria. These fifteen studies were reviewed in a similar manner and had to meet the same inclusion and eligibility criteria. See Figure 2 for a diagram of the literature selection process.

### 2.2. Data Extraction

From each study, the following information is extracted: authors, year of publication, sensor type and measured feature, sensing method, surgical procedure, tissue type (bone, soft tissue…), tissue origin (human, animal, artificial…), data analysis and decision-making procedure, accuracy, whether or not the sensor was used in combination with a robotic system, and finally, the possibility of real-time sensing. The primary outcome measures were the sensor type and sensing method. The secondary outcome measures were the decision-making procedure and the accuracy. For the goal of the sensing method or its use in surgery, often the following expressions were used: ‘tissue discrimination’ and ‘drilling/milling state identification’. In this review, we consider this as the same goal since the drilling or milling state during surgery mainly depends on the type of tissue.

## 3. Results

### 3.1. Studies Included

The literature search identified a total of 765 studies. After the removal of duplicate results, 718 papers remained for screening based on title and abstract. Based on relevance, 672 papers were excluded because of relevance, for example, they did not cover spine sensing. From the 46 remaining studies, no full text could be retrieved for one study so that 45 papers were assessed for eligibility. Finally, ten studies were rejected because they did not meet one or more of the eligibility criteria. From all the studies identified via databases through a systematic search, 35 studies were included in the review. Through searching within the references of the 35 included studies, fifteen more studies were identified based on the title and their reason for citation in the included studies. After a full review, three of those studies were excluded because they lacked relevant information on the sensor and sensing method. No full-text was available for one study and three other studies were too old. This means that seven studies were included based on reference searching, hence the total number of included studies was 43.

### 3.2. Force and Torque

Fifteen studies cover force or torque sensing during spinal surgery. A summary of the sensor types and the objectives of sensing during spine surgery is shown in Table 1. The characteristics of each study are displayed in Table 2. In three of those studies, the force data are also fused with audio data [18,19,20]. The force sensors used in those fusion studies will be listed in the current section, the vibration sensors in Section 3.3. The data fusion will also be discussed in Section 3.3.

#### 3.2.1. Related Surgical Action

Five different surgical actions or procedures were involved in the studies that discussed force or torque sensing: drilling state identification, milling state identification, intraoperative bone quality assessment, breakthrough detection during pedicle drilling and the prediction of milling depth (see Table 1). Seven studies measured the axial thrust force during drilling [21,22,23,24,25,26,27] while one study measured the torque during pedicle hole drilling [18]. One study measured the thrust force during pilot hole drilling in a vertebral tissue for tissue discrimination and then inserted a screwable dental implant while measuring the implantation torque to estimate the implant fixation [28]. Three studies measured the transverse force during the milling of the lamina [29,30,31]. With [29], this was performed to estimate the cutting depth based on a prediction model in which a set value for the milling force is related to each 0.2 mm depth of the burr. The goal of [30,31] was to perform milling state identification. One study measured the breakaway torque by loading a flanged torque meter in the pedicle until the trabecular bone succumbs to estimate the quality of the cancellous bone [32].

**Table 1:** Summary of the sensor type and function for the studies including force and torque sensing.

	Sensor Type	Load Cell	6DOF f/t Sensor	Torque Meter
Objective				
Drilling state identification		[26]	[18,19,20,22,23,24,25,27]	
Milling state identification		[31]	[30]	
Bone quality assessment		[28]		[32]
Breakthrough detection		[21]		
Prediction of milling depth			[29]	

**Table 2:** Characteristics of studies that include force and torque sensing. Use of the symbol ‘/‘ indicates that no information was found in the article.

Author and Year	Sensor	Measured Feature	Objective	Sample	Robot	Real-Time	Data Analysis	Accuracy Range
Hessinger et al. 2013 [21]	Load cell (own design)	Thrust force	Breakthrough prevention	Artificial (plywood and poly-styrene)	No	Yes	Thresholding	/
Hu et al. 2013 [22]	6DOF force/torque sensor	Force average from multiple axes	Drilling state identification	Bovine	Yes	Yes	Thresholding	/
Jiang et al. 2017 [23]	Mini40 6DOF force/torque sensor	Thrust force	Drilling state identification	Porcine	Yes	Yes	Thresholding	100%
Jiang et al. 2019 [29]	Mini40 6DOF force/torque sensor	Thrust force	Prediction of milling depth	Bovine	Yes	No	Particle swarm optimisation algorithm	/
Jiang et al. 2020 [24]	Mini40 6DOF force/torque sensor	Thrust force	Drilling state identification	Porcine	Yes	Yes	Thresholding	95%
Jin et al. 2012 [25]	6DOF force/torque sensor	Thrust force	Drilling state identification	Bovine	No	Yes	Thresholding	/
Jin et al. 2014 [18]	6DOF force/torque sensor	Drilling torque	Drilling state identification	Human	Yes	Yes	Thresholding (torque)	-
Li et al. 2021 [19]	ATI F/T sensor	Force (not specified)	Drilling state identification	Porcine	Yes	/	SVM	81–88% (fused)
Popp et al. 2013 [32]	DensiProbe	Spongy bone breakaway torque	Bone quality estimation	Human	No	Yes	/	/
Qu et al. 2021 [30]	6DOF force sensor M8128C6	Force (multiple axes)	Milling state identification	Porcine	Yes	Yes	Back-propagation ANN	92.8–100%
Rossini et al. 2020 [26]	Load cell	Thrust force	Tissue discrimination	Porcine	No	No	Thresholding	True Pos: 80% - >90% False Neg: <10–35%
Sun et al. 2020 [20]	ATI F/T sensor	Thrust force	Drilling state identification	Caprine	Yes	Yes	ANN Long short-term memory (LSTM)	88–96% (force)
Tian et al. 2014 [27]	6DOF force/torque sensor	Force (multiple axes) ->average	Drilling state identification	Ovine	Yes	Yes	Thresholding	100%
Voumard et al. 2019 [28]	Load cell M-2025 and Osstell ISQ device	Axial force and implantation torque and resonance frequencies	Bone quality estimation and implant stability estimation	Human and bovine	No	Yes	Thresholding	/
Wang et al. 2010 [31]	LC0505 piezoelectric quartz transducer	Thrust force	Milling state identification	Porcine	Yes	Yes	Thresholding	/

#### 3.2.2. Sensor Type, Sensing Method, and Purpose

Eight studies mentioned the use of a 6DOF force/torque sensor [18,22,23,24,25,27,29,30] to measure the force in the direction of the feed or the torque of the machining tool. Specifications were given for the sensor by Qu et al. who used an M8128C6 six-axis force sensor with an M8128 Interface Box (SRI, Canton, MI, USA) and by Jiang et al. who used the Mini40 force/torque sensor (ATI, Lake Orion, MI, USA). In general, such 6DOF force/torque sensors use strain gauges on a deformation object to quantify the force applied to it. The sensor is located between the drill and the robot arm for robotic spine surgery and between the drill bit bearing and the housing for handheld surgical drills. Three studies explicitly mentioned the use of strain gauges to measure the axial thrust force [21,26,28] during drilling. Hessinger et al. instrumented a self-designed deformation object with strain gauges. Rossini et al. used an unspecified load cell and Voumard et al. used the M-2025 load cell with 0.2% accuracy class (Lorenz Messtechnik GmbH, Aldorf, Germany) that was placed underneath the bone sample.

**Figure 3:**
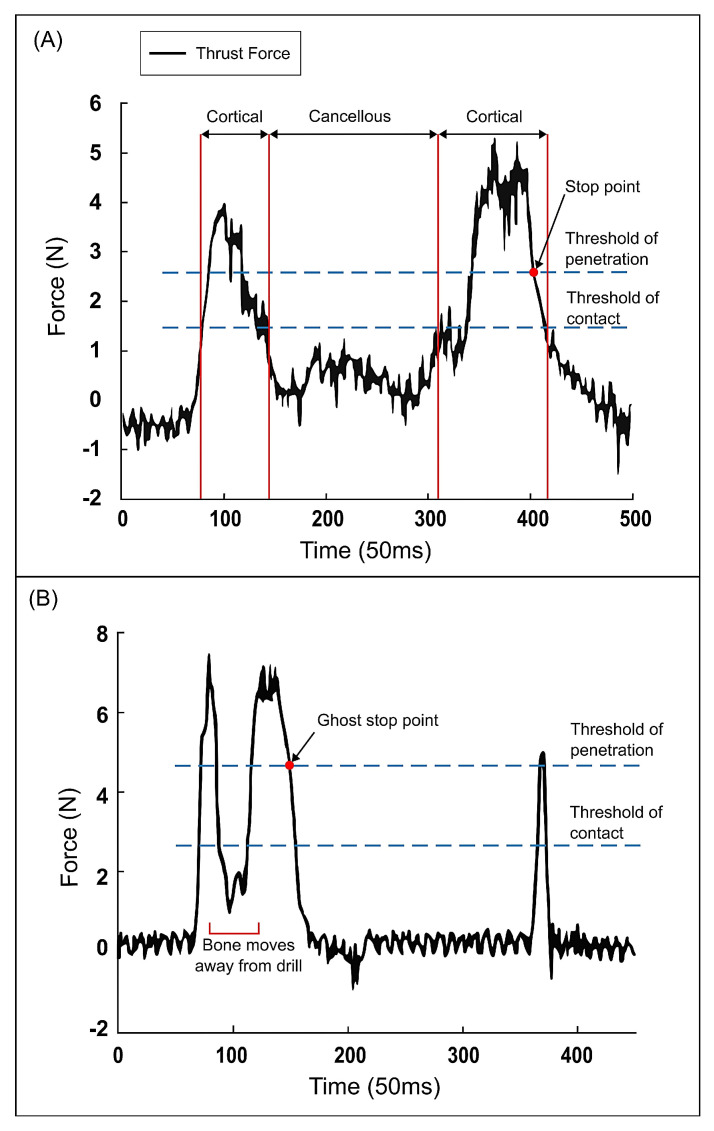
Tissue discrimination during bone drilling based on the thrust force signal. A stop point can be determined to prevent breakthrough (**A**). Faulty assignment of stop point in case of a moving bone due to, e.g., breathing (**B**). When the bone moves away from the drill, the measured thrust force drops. When the drill continues to cut through the first cortical layer while the bone moves back towards the drill, the force rises again and the system incorrectly labels the second peak as the second cortical layer. Adapted, with permission, from [23] ©[2017] IEEE.

One study made use of the LC0505 piezoelectric quartz force sensor (Lance Technologies, Copley, OH, USA) to measure the thrust force in the robot milling of the lamina [31]. The data recorded by these sensors were often used to perform thresholding on the thrust force amplitude in order to differentiate between drilling in cancellous or cortical bone. Thresholding was applied in [18,21,22,23,24,25,26,27,28,31] where, for example, Tian et al. formulated a force threshold that corresponds with drilling into the second cortical layer. The drilling process was halted immediately when the threshold was reached. However, problems can occur when the sample moves due to, for example, breathing. The feed rate changes and thus the force will fluctuate more, leading to a force profile with two peaks during the drilling of the first cortical layer [23]. A control system will falsely identify that second peak as drilling in the second cortical layer and stop the procedure (see Figure 3). When the bone is temporarily moving along with the feed of the drill, the contact force can be much lower than in a situation with stationary tissue. As a consequence, the system does not recognise the penetration of a cortical layer of bone.

Jiang et al. solved this issue by the optical motion tracking of the bone with the Polaris Vicra System (NDI, Waterloo, ON, Canada). The motion of the robot was compensated based on the real-time position of the bone such that the feed rate remained constant relative to the bone [23,24]. Rossini et al. solved a similar problem, where the force profile is inaccurate due to an irregular feed rate in manual drilling, by defining a new parameter: the average impedance (AI) [26].

(1) $$AI\left(f\right)=\frac{F(f)}{V(f)}=\frac{F(f)}{\left(2\pi fj\cdot S(f)\right)}$$

*F*, *V*, and *S* in Equation (1) represent the Fourier transforms of, respectively, the contact force between the drill bit and tissue, the velocity of the drill bit and its displacement. By creating a profile of the AI instead of the thrust force, the difference between drilling in cortical bone and cancellous bone becomes clearer and more consistent when the feed rate is not constant [26]. In [30], the force signal (after wavelet transformation to reduce noise) was used as input for a backpropagation artificial neural network (ANN) that can distinguish between milling in the outer cortical layer, the cancellous bone, the transitional layer between cancellous and cortical bone, and the inner cortical layer of the lamina. Furthermore, milling is performed by an ultrasonic bone scalpel that uses high-frequency vibrations to machine bone tissue with high impedance. Elastic soft tissue such as muscles or blood vessels is not cut by these vibrations and the bone surface after milling is smoother and flatter [30].

Finally, [32] used the DensiProbe, a tool very similar to a pedicle probe enhanced with a digital torque meter (ARI, Davos, Switzerland), to assess the bone quality in the pedicles before screw placement. The DensiProbe has three small flanges to increase the contact area at the tip which is inserted in the bone. A torque is applied until the cancellous bone succumbs. That breakaway torque is correlated with the bone quality to decide whether augmentation techniques such as the application of bone cement should be used. A strong correlation between the breakaway torque and the local volumetric bone mineral density (vBMD) was found (R = 0.90; *p* = 0.002).

#### 3.2.3. Accuracy

Six studies analysed the accuracy of the used sensing and classifying methods. Tian et al. analysed the pilot holes for pedicle screws: the drill did not penetrate the second cortical layer for all 32 holes, even for deliberately incorrectly planned trajectories [27]. The accuracy of the applied tissue recognition methods was assessed by [30] by means of force profiles that served as input for an ANN. This resulted in a recognition rate between 92.8% and 100%. Jin et al. assessed the accuracy by means of thresholding of the average drilling torque which resulted in two sets for the precision of state recognition: 67.29–74.29% and 71.87–81.88% [25]. The lower and upper values of each set corresponds to whether or not the system can differentiate between drilling in the outer and in the inner cortical layers (upper value corresponds with no differentiation). The difference between the first and the second set depends on whether, respectively, four states had to be recognised (free running of drill included) or three states (free running of drill not included). No data on the amount of test cases were available. Jiang et al. found that the success rate of detecting the correct halting point during was 60% (12/20 experiments) when no compensation for the movement of the bone was applied. When the robot compensated for sample motion, the success rate increased to 100% (20/20) [23].

#### 3.2.4. Summary

For force and torque sensing, the most commonly used sensor in this literature study is the 6DOF force/torque sensor (see Table 1). This sensor type is generally made up of strain gauges to estimate the force based on the deformation of an object. That is also the case for load cells, used in [28] and designed in [21]. One study used a piezoelectric quarts transducer to measure the feed force and one study used a digital torque meter to estimate the bone quality.

Table 2 shows that, in most of the reviewed studies regarding force and torque sensing, the thrust force is used as a main feature for analysis. That analysis is most commonly performed by simple thresholding without the aid of artificial intelligence. The typical objective in the reviewed studies is state identification during drilling or milling which aims to discriminate whether the tool is machining tissue or not and, more importantly, which tissue is being machined. A robot performed the operation in eight out of thirteen studies. This indicates that force and torque sensing is a viable approach to investigate for application in the control of an autonomous surgical robot.

### 3.3. Sound and Vibration

Twenty-one studies were found that performed sensing in spine surgery using sound and vibration. Three of those studies combined sound and vibration sensing with force sensing [18,19,20]. A summary of the sensor types and the objectives of sensing during spine surgery is shown in Table 3. The characteristics of each study are displayed in Table 4.

#### 3.3.1. Related Surgical Action

The studies using sound and vibration sensing contained five different surgical actions or procedures: drilling state identification, milling state identification, cutting state identification, breakthrough detection during bone drilling, and monitoring of bone and implant integrity (see Table 3). Eight studies from the research group of Dai et al. analysed the measurements during bone milling to recognise the tissue that is being milled [33,34,35,36,37,38,39,40]. Six studies did the same for drilling pilot holes for pedicle screw placement (PSP), drilling during laminectomy and during cervical discectomy [19,20,41,42,43,44,45]. In one study, measurements were performed during the cutting of large pieces of the vertebra such as the spinous process [46]. In two studies, acoustic emission signals (AE-signals) were analysed to determine the point during screw insertion at which the screw is overtightened and the thread in the bone is stripped or damaged [47,48]. Finally, there were also two other studies where specific pathologies in the (instrumented) vertebra were assessed by an analysis of the vibration and acoustic emissions [49,50].

**Table 3:** Summary of sensor type and function for the studies including sound and vibration sensing.

	Sensor Type	Accelerometer	Microphone	Laser Displacement Sensor	AE-Sensor
Objective					
Drilling state identification			[18,19,20,33,43,45]	[41]	
Milling state identification		[37,39,40]	[34,38,39]	[35,36,37]	
Cutting state identification		[46]			
Breakthrough detection			[44]		
Detect bone structural alterations		[50]			[47,48,49]

**Table 4:** Characteristics of studies that include sound and vibration sensing. Use of the symbol ‘/‘ indicates that no information was found in the article.

Author and Year	Sensor	Measured Feature	Objective	Sample	Robot	Real-Time	Data Analysis	Accuracy Range
Arun et al. 2014 [49]	Nano-30 miniature AE sensor	Bone AE-signal	Detect fracture occurrence	Human	No	/	/	/
Bai et al. 2021 [33]	46BE free-field microphone (GRAS, Holte, Denmark)	Sound pressure	Milling state identification	Porcine	Yes	Yes	Thresholding, statistical sample *t*-test	/
Dai et al. 2013 [41]	Laser displacement sensor LK-H082	Bone vibration (displacement)	Drilling state identification	Porcine	No	Yes	Thresholding	94.7%
Dai et al. 2015a [34]	46BE free-field microphone	Sound pressure	Milling state identification	Porcine	No	Yes	Thresholding	/
Dai et al. 2015b [35]	Laser displacement sensor LK-H082	Tissue vibration (displacement)	Tissue identification during milling	Porcine	Yes	Yes	ANN	83–100%
Dai et al. 2015c [36]	Laser displacement sensor LK-H082	Bone vibration (displacement)	Milling state identification	Porcine	Yes	Yes	Thresholding	/
Dai et al. 2016 [37]	Laser displacement sensor LK-H082 and PCB 352C33 accelerometer	Tissue vibration (displacement) and tool vibration (acceleration)	Tissue identification during milling	Porcine	Yes	Yes	SVM	76–100%
Dai et al. 2018 [39]	46BE free-field microphone and PCB 352C33 accelerometer	Sound pressure and tool vibration (acceleration)	Milling state identification	Porcine	Yes	Yes	ANN	Sensitivity: 90–100% Specificity: 82–100%
Dai et al. 2020 [40]	PCB 352C33 accelerometer	Tool vibration (acceleration)	Milling state identification	Porcine	Yes	Yes	ANN	Sensitivity:90–98% Specificity:94–100%
Dai et al. 2017 [38]	46BE free-field microphone	Sound pressure	Milling state identification	Porcine	Yes	Yes	ANN Self Organising Feature Map (SOFM)	85–95%
Guan et al. 2018 [45]	Sound detector SEN-12642	Bone AE-signal	Drilling state identification	Animal (/)	Yes	Yes	ANN	75.0–84.2%
Jin et al. 2014 [18]	Microphone	Sound pressure	Drilling state identification	Human	Yes	Yes	SVM (audio)	67–82% (audio), no fusion results
Kawchuck et al. 2009 [50]	PCB 356A35 triaxial accelerometer	Screw vibration (acceleration)	Identify structural alterations	Porcine	No	/	ANN	99.7–99.8%
Li et al. 2021 [19]	Sound detector SEN-12642	Bone AE-signal	Drilling state identification	Porcine	Yes	/	SVM	81–88% (fused)
Osa et al. 2015 [46]	TSND 121 accelerometer logger	Tool acceleration and angular velocity	Cutting state identification	Artificial (sawbones)	No	Yes	SVM	75%
Pullin et al. 2017 [47]	Pico-Z AE-sensor	Bone AE- signal	Detect stripping of screw thread in bone	Artificial (sawbones)	No	No	ANN Self Organising Map (SOM)	/
Seibold et al. 2021 [42]	Self-produced contact microphone	Bone AE- signal	Breakthrough prevention	Human	No	Yes	Convolutional neural network (CNN) classifier	93.6%
Shao et al. 2019 [43]	46BE free-field microphone	Sound pressure	Drilling state identification	Porcine	No	Yes	Thresholding, one-way ANOVA statistical analysis	/
Sun et al. 2020 [20]	Sound detector SEN-12642	Bone AE-signal	Drilling state identification	Caprine	Yes	Yes	ANN Long Short-Term Memory (LSTM)	62% (audio), 92–96% (fused)
Torun et al. 2018 [44]	ICD PX333 sound recorder	Sound pressure	Breakthrough prevention	Artificial (plywood and polystyrene)	Yes	Yes	Thresholding	100%
Wright et al. 2020 [48]	Mistras Pico piezoelectric sensor	Bone AE- signal	Detect stripping of screw thread in bone	Human	No	Yes	/	/

#### 3.3.2. Sensor Type, Sensing Method, and Purpose

Four studies from the group of Dai et al. mentioned the use of an accelerometer [37,38,39,40]: they used a single-axis accelerometer PCB 352C33 (PCB Piezotronics, Depew, NY, USA) mounted on the power tool to identify the milling state of the tool based on its vibrations. The denser cortical bone causes larger vibrations in the tool than the cancellous bone due to its higher resistance against machining. The signal can be decomposed through wavelet packet transform (WPT) into various narrow frequency bands which all contain one harmonic of the spindle frequency. Machining in the dense cortical bone leads to larger harmonic amplitudes such that analysis of these harmonics (or the WPT energy) can be used for the identification of several drilling or milling conditions. In other research, the wavelet transform technique has been shown to be a good predictor of structural integrity and even the location of damage in a structure [51]. Milling state identification was performed by Dai et al. with support vector machine (SVM), which is a machine-learning algorithm for data classification in [37] or an ANN in [38,39,40]. The TSND 121 (ATR-Promotions Inc., Kyoto, Japan), an acceleration and gyro sensor attached to the proximal end of the tool, was used in [46] to measure the axial acceleration and angular velocity of a power cutting bur. In one case, the accelerometers were not attached to the tool but to screws inserted into the vertebra [50]. In that work, the researchers used triaxial accelerometers type 356A35 (PCB Piezotronics, Depew, NY, USA) to identify the presence, location and magnitude of the structural alterations (healthy vertebrae, interlinked vertebrae, vertebral disc stabbed by scalpel and disc transection) within the spine. A neural network was trained to recognise the different alterations.

In five studies from the same group from Nankai University (Tianjin, China), a microphone was used to capture the sound of the machining process [33,34,38,39,43]. For all five studies, the employed microphone was a 46BE free-field microphone (GRAS, Holte, Denmark) attached at a distance of 5–20 cm from the tool by means of a metal clamp that was fixed to the tool or the robot arm. The wavelet method for extracting the harmonic amplitudes was used and the state identification was performed with an ANN in [38,39] or by the thresholding of the harmonic amplitudes or the WPT energy in [34,43]. For Bai et al., the sound was recorded for three milling states: milling the lamina cancellous bone, milling the ventral cortical bone and penetrating the ventral cortical bone. For each of those states the magnitude of the frequency spectrum at 1, 2, 3, 4 and 5 kHz was analysed and a statistical (independent sample) *t*-test was performed to correlate the ‘fingerprint’ of the frequency spectrum with the milling state [33].

**Figure 4:**
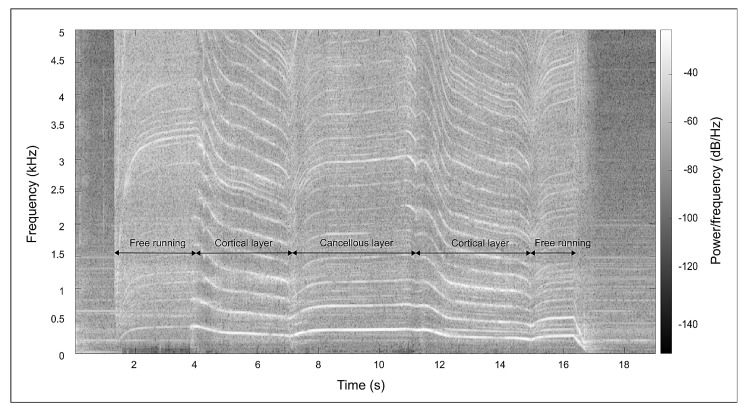
Spectrogram to differentiate tissue types during bone drilling and to detect critical moments during the process. In the first stage, the drill is off, then the drill is turned on and runs free without a load. Next, the drill enters the first cortical layer, slowing down so that the frequency drops. It then drills through to the cancellous bone and accelerates again due to a lower resistance. Then, the drill cuts into the second cortical layer with a similar effect on the frequency as the first cortical layer and finally breaks through it. The drill then experiences less or no load anymore and accelerates. Finally, the drill is turned off again. Adapted, with permission, from [44] ©[2018] IEEE.

In one study, the researchers used a low-cost, self-produced contact microphone that was connected to the skin with a kinesiology tape to detect AE-signals during bone drilling [42]. The measurements were recorded in a log-mel spectrogram which was used as input for a convolutional neural network (CNN) classifier. Torun et al. used spectrograms to visualise the audio signals measured with a Sony ICD PX333 sound recorder (Sony, Tokyo, Japan) [44]. They noticed that the frequency of the signal dropped as the drill was about to break through a cortical layer of bone (see Figure 4). Based on this, they defined a new parameter, the MNMD, which is a combination of the mean (MN) and median (MD) frequency at each time instance in the spectrogram. If the MNMD fell below a certain threshold value, it was concluded that the breach was imminent [44].

Four studies from one research group mentioned the use of the LK-H082 laser displacement sensor (Keyence, Osaka, Japan) [35,36,37,41]. This sensor emits a laser beam to the sample which is then reflected back to the sensor. As the sample moves or vibrates, the angle with which the laser is reflected back from the sample to the sensor changes and this change is equivalent to the displacement of the sample relative to the sensor. Similar methods to perform state identification used for the accelerometer or microphone were applied for the laser displacement sensor. These methods encompass the use of an ANN [35], an SVM classifier [36], or thresholding of the harmonic amplitudes [37,41].

Four studies describe the use of an acoustic emission (AE) sensor [19,20,45,49]. In the strict sense, acoustic emission sensors are piezoelectric sensors, similar to accelerometers, that measure very high frequency signals from travelling stress waves in a structure. That type of sensor was applied by Arun et al., using the Nano-30 miniature AE sensor (Mistras, Princeton, NJ, USA), attached to the lumbar vertebrae to detect the fracture signal during impact loading on the lumbar spine in the caudal direction [49]. Two studies used AE-sensors in combination with load cells (which were not specified) to detect the overtightening of orthopaedic screws. Ref. [47] used the Pico-Z (Pancom, Fenstanton, UK) and [48] used the Mistras Pico piezoelectric sensor (Physical Acoustics, West Windsor Township, NJ, USA) to detect AE-signals. Both studies concluded that stripping, and thus damage to the thread in the bone, occurred when the accumulated AE-energy continued to increase during tightening while the axial load of the screw did not. In a real surgery, it is impossible to place a load cell between the bone and the screw. Therefore, Wright et al. investigated the correlation between the recorded AE energy and both the bone mineral density and the stripping force. A high positive correlation was found [48]. In [19,20,45], however, the researchers used the SparkFun Sound Detector SEN-12642 (SparkFun, Niwot, CO, USA) which consists of a microphone on a printboard. It can be regarded as an AE-sensor in the broad sense because it measures very high frequency waves travelling in the air, but not in the strict sense because no structural pressure waves are measured. Even though Guan et al., Sun et al., and Li et al. used the term ‘acoustic emission signal’, we will categorise this sensor as a microphone in Table 3 due to the fact that the sensor measures sound pressure in the air (in the audible frequency range) and not the high-frequency stress waves in the structure. The sensor used in [45] was mounted 5 cm above the tip of the drill bit and measured in the range from 10 to 15 kHz. State identification was performed through curve fitting with a function of the form:

(2) $$y=a\cdot e^{bx}\cdot sin\left[\left(2\pi +cx\left(1-x\right)\right)x^{d}\right]$$

where *a*, *b*, *c*, and *d* are the coefficients that are adjusted to fit the measured data best. The coefficients of the obtained function are used as input for an ANN that can then identify, based on the different combinations of high and low values for each coefficient, the different bone layers encountered during drilling. The same sensor was applied by [19,20] to perform the differentiation of drilling in cortical bone, in cancellous bone, or in the transition region between those two layers. The thrust force was measured simultaneously with an ATI force sensor (ATI Industrial Automation, Apex, NC, USA) to perform state recognition (drilling in cortical bone, cancellous bone, and the transition region) based on the force amplitude and the rate of change in the force signal. A fusion of state recognition with the audio signal and state recognition with the force signal was used to reach higher recognition rates. Recognition based on force features could accurately discriminate the cortical from the cancellous bone whilst having difficulties in the transition region. Recognition based on the audio features could better identify the transition region but proved more difficult to distinguish the cortical from the cancellous bone.

#### 3.3.3. Accuracy

Twelve studies assessed the accuracy of the tested measurement system. With a laser displacement sensor, Dai et al. were able to achieve a success rate of 94.7% (18/19 breakthrough experiments) in detecting the thrust-in point of the drill in the inner cortical layer [41]. The same research group continued to perform more detailed accuracy assessments in later studies. In [35,38], the results showed there was no fundamental loss of discrimination accuracy when a microphone or laser displacement sensor was moved further away from the sample. In [37], the importance of milling with the same milling parameters during the testing and during training of an ANN was shown. When the feed rate was increased from 0.5 mm/s during training to 1 mm/s during the testing of the ANN and the spindle speed was increased from 12,000 rpm to 30,000 rpm, the accuracy dropped by 11% for measurements on the drilled vertebra and by 24% for measurements on an adjacent vertebra. In [39], both data from an accelerometer and a microphone were used to identify which tissue was being milled. The highest accuracy was achieved when the correlation coefficient between the harmonics of both signals was fed into the ANN. When the signal from the accelerometer and the microphone was fed separately into the ANN, the accuracy of tissue discrimination was lower. The same phenomenon was observed by Sun et al. [20]. For pedicle screw placement (PSP), the force and sound were measured during pilot hole drilling. The identification rate of the inner transition region between the cancellous and cortical bone was 88% (in 100 experiments) for the analysis of the force signal and 62% (in 100 experiments) for the analysis of the audio signal. When both signals were combined and fused detection was applied, in 92 out of 100 experiments, the transition region was correctly identified [20]. Dai et al. also assessed the accuracy of their system to perform tissue discrimination in the vertebra at different signal-to-noise ratios (SNRs). Intuitively, the best accuracy was reached when no artificially added noise was present in the measurements. Considerably good results were found when the SNR was equal to 1. In that case, the sensitivity dropped by a maximum of 8% [40]. This was possible because analog-to-digital converting and encoding was performed by a microchip-based platform near the accelerometer that can prevent the contamination of environmental noise. In [42,44], breakthrough detection based on features from a spectrogram resulted in accuracies well above 90%. When the window length of the spectrogram, which determines the number of samples and acquisition time, was lowered (e.g., from 100 ms to 25 ms), the accuracy dropped by 9%. This happens because the system can provide a detection result faster in a shorter window but has less information that can be used for feature extraction [42]. Finally, Kawchuck et al. reported that decision making based on the data of a single axis (the dorsoventral axis) performs similarly compared to when the data of all three axes of the accelerometer are used [50].

#### 3.3.4. Summary

For sound and vibration sensing, there is not one type of sensor that is reported drastically more often than other sensors (see Table 3). In five studies, accelerometers are used to measure vibrations. Four of those studies applied the sensor to the tool to assess the dynamic features during the machining of the vertebra and one applied it to screws of instrumented vertebrae. There is only one out of those five studies in which a triaxial accelerometer is used but the authors conclude that a monoaxial accelerometer performs equivalently [50]. Acoustic emission sensors are used in three studies. Four studies mention the use of a microphone, placed anywhere between right next to the drill bit and up to two metres away from the drill, and in four studies, a laser displacement sensor is pointed at the tissue to measure its vibrations. Both the microphone and the laser sensor have the advantage that they are non-contact sensors and that they can stay out of the sterile area in the operating room. In the case of sound and vibration sensing, the most frequently applied method of data analysis in this review is by means of an ANN. Table 4 shows that different types are used, such as the convolutional neural network (CNN), which is commonly applied to analyse visual imagery, or the self-organising map, which excels at representing the relationship between the values of high-dimensional datasets. Data fusion from different sensors such as accelerometer and microphone [39] or force sensor and microphone [19,20,45] has shown to improve the performance of milling or drilling state identification because they perform better at different events or states such that through fusion, and the monitoring of the entire procedure is enhanced. In nine out of eighteen studies, a robot performed the drilling or milling operation from which the data were collected.

### 3.4. Bioelectrical Properties

Ten studies were found that measured the bioelectrical properties such as the impedance of the tissues in and around the vertebra to monitor unintentional tissue damage. A summary of the sensor types and the objectives of sensing during spine surgery is shown in Table 5. The characteristics of each study are displayed in Table 6.

#### 3.4.1. Related Surgical Action

Four different surgical actions were identified in the studies that measured bioelectrical properties: cutting state identification, breakthrough detection during drilling, detection of screw malpositioning and detection of spinal motor tract injury (see Table 5). The related surgical procedures were pedicle screw placement (PSP), posterior longitudinal ligament (PLL) resection, and spinal cord decompression. Eight studies focused on PSP, among which three studies monitored the breach of the pedicle wall by tissue discrimination during the probing of the pedicle canal [52,53,54]. Two of eight studies checked the pedicle wall in an empty pedicle canal after probing to detect whether the penetration of the cortical wall had happened [55,56]. Three of eight studies measured the screw after placement to detect whether the screw had breached the cortical wall [57,58,59]. One study investigated tissue discrimination in the PLL-reSection [60]. One study investigated the detection of the spinal motor tract injury during spinal cord decompression surgery [61].

**Table 5:** Summary of sensor type and function for the studies including the sensing of bioelectrical properties.

	Sensor Type	Conductivity Measurement Device	EMG Probe	LCR Sensor
Objective				
Cutting state identification				[60]
Breakthrough detection		[52,53,54]		
Detection of screw malpositioning			[55,56,57,58,59]	
Detection of spinal motor tract injury			[61]	

**Table 6:** Characteristics of studies that include sensing of bioelectrical properties. Use of the symbol ‘/‘ indicates that no information was found in the article.

Author and Year	Sensor	Measured Feature	Objective	Sample	Robot	Real-Time	Data Analysis	Accuracy Range
Bolger et al. 2007 [52]	PediGuard conductivity measurement device	Electrical conductivity	Breakthrough prevention	Human	No	Yes	Thresholding	96–100%
Chaput et al. 2012 [53]	PediGuard conductivity measurement device	Electrical conductivity	Breakthrough prevention	Human	No	Yes	Thresholding	97.5%
de Blas et al. 2012 [57]	12 mm monopolar needle	Electromyography activity	Detection of screw malpositioning	Human	No	Yes	Thresholding	34%
Donohue et al. 2008 [55]	Ball-tipped probe	Electromyography activity	Detection of screw malpositioning	Human	No	Yes	Thresholding	57.9–94.7%
Montes et al. 2012 [58]	Monopolar needle electrodes	Electromyography activity	Identify pedicle wall integrity	Porcine	No	Yes	Thresholding	/
Rodriguez et al. 2008 [59]	Subdermal needle	Electromyography activity	Detection of screw malpositioning	Human	No	Yes	Thresholding	54.5%
Samdani et al. 2011 [56]	Letz Ball Electrode, JO-5 style needle, 12 mm needle electrodes	Electromyography activity	Detection of screw malpositioning	Human	No	Yes	Thresholding	17%
Shao et al. 2019 [60]	LCR meter 4285A	Bioelectrical impedance	Tissue discrimination	Porcine	No	yes	Thresholding	/
Skinner et al. 2009 [61]	/	Electromyography activity	Detection of spinal motor tract injury	Human	No	Yes	Thresholding	PPV: 80% NPV: 95%
Zeller et al. 2009 [54]	PediGuard conductivity measurement device	Electrical conductivity	Breakthrough prevention	Human	No	Yes	Thresholding	100%

#### 3.4.2. Sensor Type, Sensing Method and Purpose

**Figure 5:**
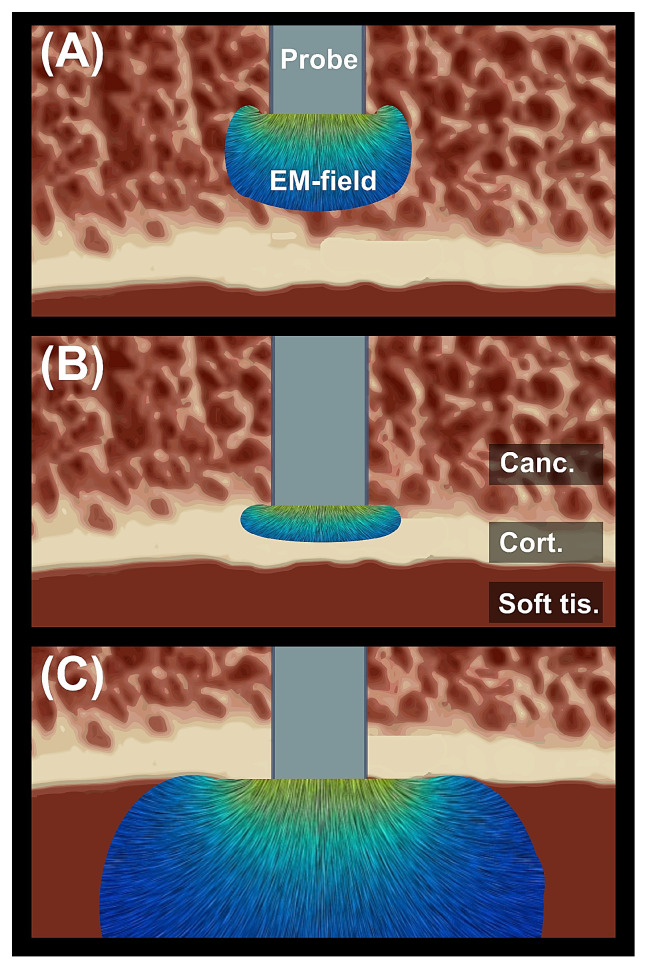
A probe that measures the conductivity of the surrounding tissue to differentiate the tissue type. Visualisation of probing cancellous bone (**A**), cortical bone (**B**), and soft tissue (**C**). Soft tissue (and particularly blood) is more conductive than bone tissue. In this figure, the spread of the electromagnetic field is a visual representation of the conductivity of each tissue.

The three studies that monitored pedicle perforation during probing all used the same sensor: the PediGuard electrical conductivity measuring device (Spineguard, Vincennes, France) [52,53,54]. It is a pedicle probe which measures the electrical conductivity of the tissue in the immediate proximity of the tip of the probe and emits an auditory feedback signal proportional to the measured conductivity at the tip. Cortical bone has a different conductivity compared to cancellous bone or soft tissue. The electromagnetic field that is emitted by the probe will thus change in the vicinity of a border between two tissues. That change is detectable and feedback can be given to the surgeon when the probe is approaching or puncturing the pedicle wall (see Figure 5). The surgeon aims for the auditive feedback signal to remain constant at the signal level of cancellous bone throughout the operation. Otherwise, when the feedback signal changes, the redirection of the probe can be attempted. One study used the inductance–capacitance–resistance (LCR) meter 4285A (Agilent Technologies, Santa-Clara, CA, USA) to discriminate between seven types of tissues [60]. By applying a known current (0.1 mA) between the two electrodes of the sensor and measuring the resulting voltage between the electrodes, the electrical impedance could be calculated. The statistical analysis of the impedance amplitude and phase of each tissue group proved to be significantly different for the different tissue types. This, according to the authors, indicates the potential of bioelectrical impedance to provide real-time tissue differentiation.

In six studies, electromyography (EMG) was performed to detect the occurrence of pedicle breach or the injury of soft tissue (see Table 5). Monopolar electrode needles are inserted in the muscles surrounding the vertebra to measure the electric potential generated by the muscle cells and serve as an anode in the measurement system. Low signals indicate an abnormality and thus indicate that the screw malposition has occurred. Examples of such needles are: the 12 mm monopolar needle and the JO-5 style needle (Viasys, Conshohocken, PA, USA) used in [57,59], monopolar needle electrodes by Ambu (Ambu, Ballerup, Denmark) used in [57] and stainless steel 12 mm needle electrodes by Medtronic-Xomed (Medtronic, Fridley, MN, USA) used in [56]. The stimulation and monitoring of motor-evoked potentials is performed by Samdani et al. using specific control equipment such as the Xltek Protektor (Natus Medical Incorporated, Pleasanton, CA, USA) or the Keypoint equipment (Medtronic Dantec Medical, Skovlunde, Denmark), which also contains the cathode that can be connected to the pedicle screw [56].

Two studies that used EMG technology to detect a breach in the pedicle track before the placement of the screw used a ball probe to stimulate the pedicle track: [55] used the Xomed ball-tipped probe (Medtronic, Fridley, MN, USA), and [56] used the Letz Ball Electrode (Utah Medical Products Inc., Midvale, UT, USA). These probes serve as cathodes of the system. All the studies that investigated EMG discriminated between soft tissue penetration and faultless operation by defining a threshold for the current that was applied to the screw. If the evoked EMG appeared at a low current threshold, for example, through th the unintentional movement of the lower limbs, it meant that the current could easily flow to the soft tissue and the breach of the pedicle wall was likely. The current threshold when electrical stimulation was performed on the pedicle screws ranged from 6 mA in [56,59] to 8 mA in [57] to determine with certainty that the violation of soft tissue had occurred. When stimulation was performed in the pedicle track or pilot hole by a ball-tipped probe, Donohue et al. found a threshold of 15 mA below which screw malpositioning was a certainty [55]. For values higher than the thresholds in each of these studies, there was still the possibility that screw malpositioning had occurred but it could not be determined with certainty based on the proposed methodology.

#### 3.4.3. Accuracy

Nine studies assessed the accuracy of the tested measurement system. In [52], the performance of the PediGuard tool was compared to the standard technique for PSP. Bolger et al. found that the standard technique led to 43% (10/23) of pedicle screws being well placed and the PediGuard tool led to 96% (22/23) of screws being well placed. In a second set of experiments in the same study, 100% accuracy (41/41) was achieved. Similar results were found in [53,54]. Furthermore, [60] tested the LCR-meter and reported that, in 60 sets of measurements, there were no cases where a tissue could not be discriminated either in amplitude or phase of the impedance signal. For the studies that investigated EMG, the detection accuracy is determined by the number of screws that were malpositioned (verified by postoperative CT imaging) and that fell below a certain threshold value of the stimulation current for the evoked EMG to emerge. The detection rate of the malpositioned screws varied from 17% to 54.5% [56,57,59]. Those rates account for the screws or pilot holes for which the stimulation current was so low that there was no doubt about the presence of a breach. There also exists a grey zone for the stimulation current, often between 6 and 10 mA, where the distinction between breach and no breach of the cortical wall is not clear [59]. Then, no conclusion can be made based on the EMG measurement method. Furthermore, Montes et al. showed that the current threshold depends not on the integrity of the cortical wall but rather on the distance between the screw and the spinal cord [58]. In [55], 94.7% (18/19) of cases where the drilling of the pilot hole breached the cortical wall could be identified by probing the pedicle canal with the electrode. When, in the same study, the electrode was attached to the screw to detect a breach after screw placement, 57.9% cases of malpositioning were detected. Finally, Skinner et al. found a positive predictive value of 80% for muscle-derived Transcranial Electrical Motor Evoked Potential (TCE MEP) to detect the motor tract injury and predict post-operative worsened motor function. The negative predictive value for the preserved MEP to predict intact motor function was 95% [58].

#### 3.4.4. Summary

For the sensing of the bioelectrical properties, most studies in this review investigated electromyography (see Table 5). Since sensing based on electromyography cannot distinguish between cortical and cancellous bone and cannot prevent the injury of soft tissue but can only detect it once it has happened, the measurement of EMG is not suitable for real-time sensing in spine surgery to prevent injury. As can be seen in Table 6, the performance of EMG measurements to detect screw malpositioning was generally poor. Only the probing of the pedicle track proved accurate in predicting which screw paths would lead to a breach [55]. Montes et al. even showed that the decision threshold is subject to greater influence by the distance between the screw and the soft tissue than by the integrity of the cortical wall [58]. An electrical conductivity sensor was assessed by three studies. The breach of the pedicle canal during the preparation of a pilot hole for a pedicle screw could consistently be avoided [52,53,54]. An LCR-meter can be used to discriminate between several tissue types in the spine based on the electrical impedance [60]. None of the included studies made use of a robotic system to perform the operation.

### 3.5. Overall Results

**Figure 6:**
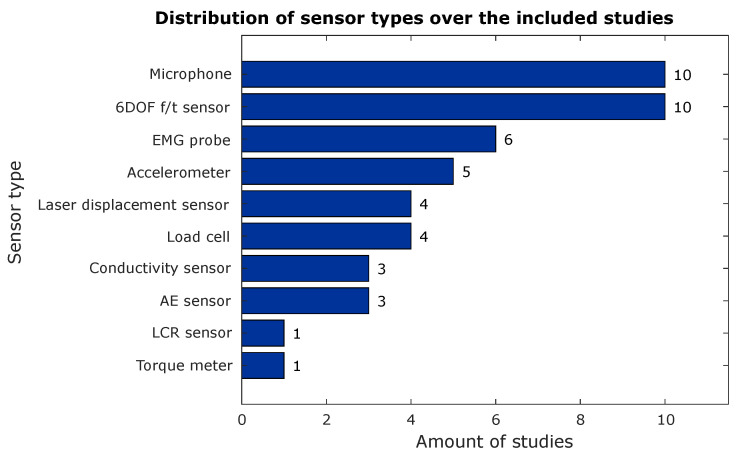
Distribution of the sensor types over the included literature, displayed in a bar plot. The length of each bar represents how many studies investigated the listed sensor types and their applicability in spine surgery.

**Figure 7:**
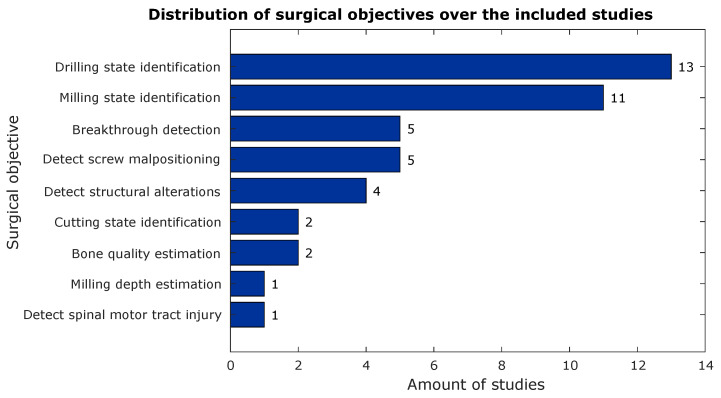
Distribution of the surgical objectives over the included literature, displayed in a bar plot. The length of each bar represents how many studies investigated a sensing method for the listed surgical action.

When all included studies are taken into account, only nine sensor types could be distinguished. The most commonly used sensors were microphones and 6DOF f/t sensors (see Figure 6). Coincidentally, there were also nine unique surgical objectives or actions distinguishable in the gathered literature. The most common objective of using the described sensor in spine surgery was to perform state identification during machining (drilling or milling) of the vertebral bone (see Figure 7). More specific or dedicated surgical actions than ‘state identification’ were represented much less often in the literature.

**Figure 8:**
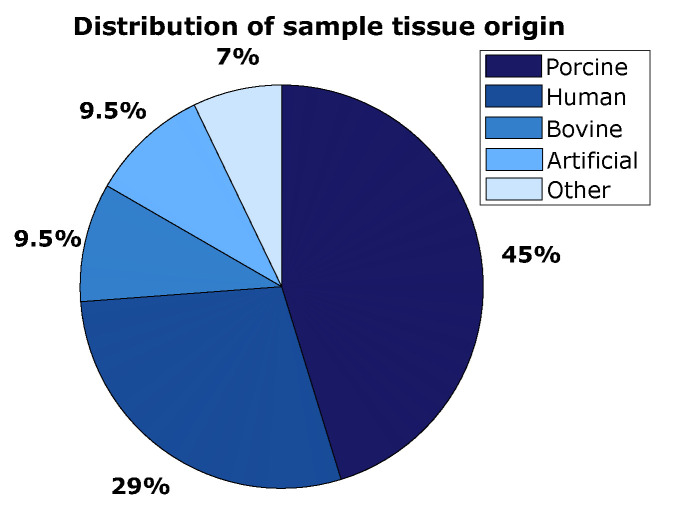
Distribution of the origin of the samples that were used for experimental work in the included literature, displayed in a pie chart. The samples labeled as ‘other’ include ovine tissue, caprine tissue, and non-specified animal tissue.

The pie chart in Figure 8 shows that almost 75% of all included studies use either porcine or human tissue to test their sensing methods. The samples labeled as ‘other’ include ovine tissue, caprine tissue and non-specified animal tissue. Over 90% of all samples consisted of biological tissue. The artificial samples were either a sandwich structure of polystyrene and plywood that resembles the layered structure of bone, or either Sawbones, a commercially available bone mimicking composite consisting of short fiber-filled epoxy resin and polyurethane (Sawbones, Malmö, Sweden).

**Figure 9:**
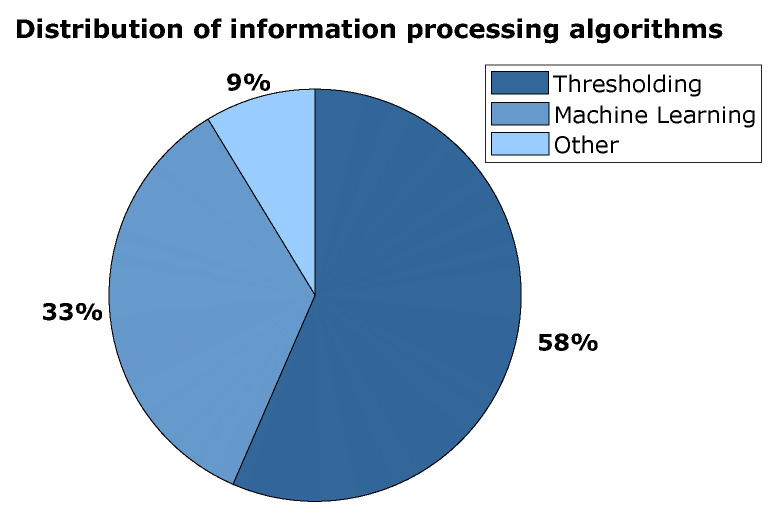
Distribution of the type of data processing and decision making performed in the included literature, displayed in a pie chart. The slice labelled as ‘other’ includes a statistical analysis that correlates bone quality to the torque required to break the trabecular bone with a dedicated tool, a particle swarm optimisation (PSO) algorithm and two algorithms that were not disclosed or clarified in their respective studies.

**Figure 10:**
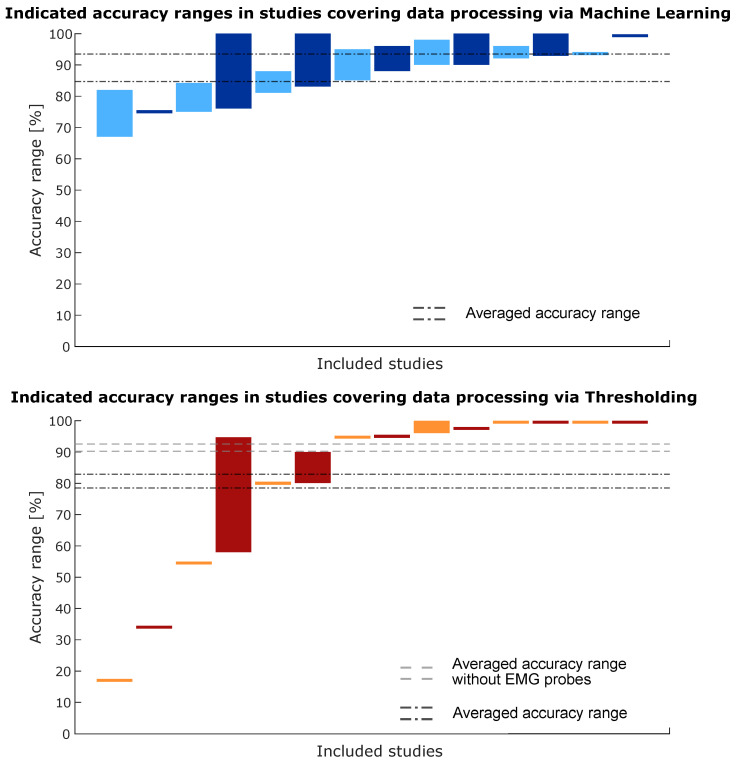
Compilation of all provided accuracy ranges in the included literature. The upper chart displays the accuracy ranges of the studies that used machine learning for data processing and decision making. Each bar represents the accuracy range of one study. The bottom chart displays the accuracy ranges of the studies that used thresholding. The dashed lines show an ‘average range’ feature that can be used for qualitative comparison between the upper and lower chart and is derived by taking the average of the maximum values and the average of the minimum values of all accuracy ranges within that chart. The alternating colours of the ranges are solely for enhanced contrast between neighbouring bars.

Important when dealing with sensors is not only the hardware but also the way in which the gathered sensor data are processed and how decisions are made based on the data. Simple thresholding of the recorded signal based on a pre- or intraoperatively determined threshold was the most common procedure in the included literature (see Figure 9). The slice labelled as ‘other’ includes a statistical analysis that correlates bone quality with the torque required to break the trabecular bone with a dedicated tool, a particle swarm optimisation (PSO) algorithm and two algorithms that were not disclosed or clarified in their respective studies. Figure 10 compiles the accuracy ranges as indicated by all studies that provide a measure of accuracy for their investigated sensing method. The provided accuracy by studies that use machine learning for data processing and decision making is compared to the provided accuracy of studies that use thresholding. It is very difficult to make a statistical analysis or comparison between the accuracy ranges of different studies, often obtained in very diverse ways. Therefore, a feature for qualitative comparison is displayed on the graphs by means of two dashed lines that represent the average maximum value and average minimum value in all accuracy ranges. It gives an idea of the central tendency of the accuracy ranges and of the width of the accuracy ranges. This ‘averaged range’ will be high on the graph if the accuracies are high over all ranges and the width of the ‘averaged range’ will be larger if the majority of the accuracy ranges are also wide. In the bottom chart of Figure 10, a second ‘averaged range’ is shown, which is calculated similarly to original ‘averaged range’ but without the accuracy ranges of the EMG probes (the first four bars on the left) as they were from a much lower order of magnitude than all other investigated sensors. Decision making based on a machine learning algorithm and based on thresholding both have an ‘average accuracy range’ around 90% when the EMG probes are excluded.

**Figure 11:**
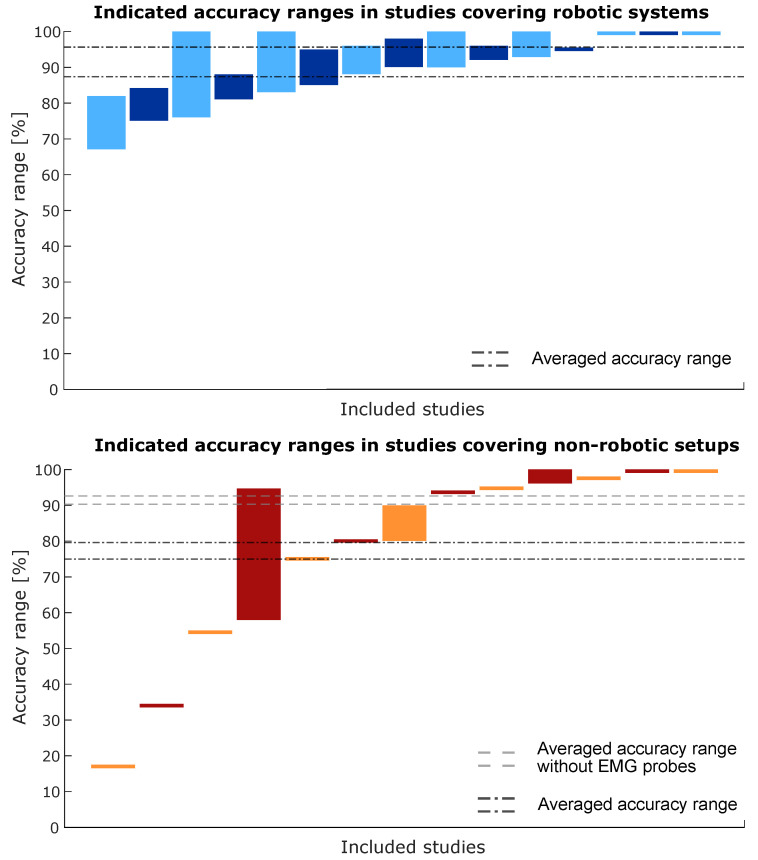
Compilation of all provided accuracy ranges in the included literature. Each bar represents the accuracy range of one study. The upper chart displays the accuracy ranges of the studies that used a robotic system. The bottom chart displays the accuracy ranges of the studies that used a non-robotic setup. The dashed lines show an ‘average range’ feature that can be used for qualitative comparison between the upper and lower chart and is derived by taking the average of the maximum values and the average of the minimum values of all accuracy ranges within that chart. The alternating colors of the ranges are solely for enhanced contrast between neighbouring bars.

A similar set of graphs is shown in Figure 11 but compares the accuracy ranges of studies performed with a robotic system to the accuracy ranges of studies performed with a non-robotic setup. The same issue arises in the bottom chart where the studies investigating EMG probes (represented by the first four bars on the left of the bottom chart) have much lower accuracy ranges and skew the ‘average range’ for the non-robotic setups. When the EMG probes are excluded, an ‘average accuracy range’ of approximately 90% is visible for studies covering robotic systems and studies covering non-robotic setups.

## 4. Discussion

This systematic review was performed to create an overview of the state of the art in non-visual, non-radiative spine sensing, constrained to measuring forces and torques, vibrations and sound and bioelectrical properties. The objectives of this review are to provide an overview of the state-of-the-art in sensing technologies in spine surgery, to identify trends in the use of certain sensors or in data processing and to investigate whether there are research gaps for future developments in the research field.

Most of the discussed sensors were tested on human or animal vertebrae and do not hinder the actions during spine surgery. The most commonly used animal substitute for human vertebrae were porcine samples, typically because of their anatomical likeness. However, there are still significant differences both anatomically and biomechanically between human and porcine vertebrae [62]. All force and torque sensors that are described in the studies were integrated in the tool or robot such that there was no interference or contact with the sample, except in the study by Voumard et al. where the sensor was placed under the tissue [28]. A similar integration in instrument or robot is possible for accelerometers and conductivity or bioelectrical impedance sensors to avoid any hindrance in and around the sterile field and for ease of use [37,39,40,46,52,53,54,60]. In the investigated studies, sensors that measure bioelectrical properties have never been used in combination with a robotic system. This shows potential for future studies on robot control by the feedback of bioelectrical impedance sensing. The measurement of an EMG-signal is deemed not suitable for the goal of autonomous robotic spine surgery because, in many cases, the signal is only measurable when the penetration of the muscle tissue has already happened. On top of that, the detection rate of malpositioned screws that breached into soft tissue was between 17 and 95% which is not consistently reliable. More specifically, in more than half of the studies that assessed the detection rate with EMG signals, the correct identification of screw malpositioning fell below 60%.

For sound and vibration sensing, there are many non-contact sensors such as microphones or laser vibrometers available that avoid the presence of the sensor in the sterile area surrounding the patient. However, the surgeons’ actions might be hindered because complete silence is required or because the presence of the surgeons’ hands and arms can block the laser beam. Accelerometers and AE-sensors have also been attached to the sample or patient to detect events such as vertebral fractures or drill breakthrough [42,49,50]. Seibold et al. showed that this does not necessarily have to be an invasive method by acquiring clear signals from contact microphones taped to the skin surface of the cadaver. That non-invasiveness makes the measurement method more applicable in spine surgery compared to when the sensor is directly attached to the bone.

From all investigated setups, only two sensors are commercially available as a finalised system in which data processing and decision making are integrated. These are the Ostell ISQ device used by Voumard et al. [28] and the Pediguard probe used by Bolger et al., Chaput et al. and Zeller et al. [52,53,54]. This should in fact happen for more setups found in this literature review. Having a finalised system with built-in functionalities for feedback to the surgeon improves the usability and also eases the combination with other sensors. The output of their validated algorithms can readily be incorporated as an input for a global inference network of a multisensor setup.

The main objective in the majority of included studies is to obtain a sort of state identification during a surgical action. This includes the ability to recognise in which type of tissue the tool is operating and to detect specific events such as the first contact with a certain tissue or the breakthrough of a cortical bone layer. For force and torque sensing, that is most commonly done by thresholding the thrust force (see Table 2). In the case of measuring the bioelectrical properties, the thresholding of the signal (conductivity, bioelectrical impedance, EMG) is performed for all studies. For the studies on sound and vibration, artificial intelligence was applied in the majority of cases to analyse the data and identify the operating state. It is very important that the situation in which the signals for training an ANN or a machine learning model are obtained, is similar to the situation in which the system will finally operate. Dai et al. concluded that if this premise is not met, the success rate of the state identification may drop as much as 24% [37].

Overall, the accuracies mentioned in the reviewed studies were high, with many cases reporting detection or (correct) classification rates of more than 80%. In general, the accuracy was lower when several types of soft tissue such as muscles and the spinal cord had to be identified. For solely bony tissues, the classification rates were over 90% for 16 out of 22 studies that mentioned the accuracy or performance in bony tissues. From Figure 10, it can be seen that there is a distinction between the accuracy ranges when machine learning is applied and when thresholding is applied. This is, however, only the case when the studies investigating EMG probes (represented by the first four bars on the bottom chart) are included because they performed generally worse than all other sensors in this review. When the EMG probes are excluded, the accuracy ranges are similar for both types of information processing. The accuracy ranges are much smaller in the case of thresholding but this is because that typically concerns a binary problem (threshold reached or not reached) which can be evaluated by one accuracy value. Machine learning, on the other had, was often applied for tissue classification which results in each tissue having its own classification accuracy, and thus, potentially large accuracy ranges within one study. Similar conclusions can be drawn from Figure 11. Robotic and non-robotic setups perform similar over the included literature when the studies investigating EMG probes are not taken into account. Interestingly, it appears from Figure 10 and Figure 11 that most studies covering robotic systems apply some sort of Machine Learning for data processing and decision making. The studies covering non-robotic setups mainly apply thresholding algorithms for decision making.

Three studies performed a combination of force and vibration sensing [18,20,39]. The main conclusion for these studies is that tissue discrimination based on a combination of both signals performs better than tissue discrimination based on the signals separately but synchronous data acquisition is essential. A dynamic signal analyser such as in [39] can be used to assure synchronous acquisition. No clear difference was found between the performance of data analysis by manual thresholding or by using artificial intelligence. Both methods are relevant for real-time feedback in surgery. In most of the studies, the system was able to perform real-time detection or classification with the longest mentioned delay lasting up to 100 ms [42]. Some studies did not mention a specific time value but they proved that the system was able to stop the tool before a critical moment (e.g., breach through a cortical bone layer into soft tissue) which we consider sufficient to be categorised as real-time decision making and control.

A final remark on the accuracy relates to how the respiration of the patient influences the fidelity of state identification. This problem was brought to attention in the literature by Jiang et al. where it was shown that the success rate of detecting the inner cortical wall and stopping the drill was increased from 60 to 100% when the compensation of the drilling feed for the respiration was applied [24].

### 4.1. Limitations

The decision concerning which literature to include in a review can never be performed in a completely objective manner. However, there are methods such as the PRISMA guidelines that were followed in this systematic review to minimise bias by introducing a systematic and reproducible strategy to search for and select scientific literature. In our review, the eligibility criteria limited the literature to studies that are written in English and published no earlier than 2005 in a journal with a Q-index of Q1 or Q2. It is likely that a large group of studies has been excluded in this way and some sensors or sensing methods were not identified because of this. Nevertheless, in this review, we aimed to outline the state-of-the-art and thus only recent papers, starting from 2005, were included. The PRISMA guidelines advocate to only include high-quality research in one’s review but no strict definition is given of what can be considered ‘high-quality research’ so we decided to only include studies from journals having a SJR score with a Q-index of Q1 or Q2.

Care should be taken when interpreting the results relating to the accuracy of the sensors in this review. The experimental sample size in each study varied widely, and for many studies, the accuracy was derived from a small number of measurements. Furthermore, Figure 10 and Figure 11 should only be qualitatively assessed. The data that each accuracy range of each study is based on are so different from one another that any statistical analysis would be very tedious. The figures are meant to provide a visual representation of trends and tendencies in the literature.

### 4.2. Future Developments

As this systematic review tries to provide a vantage point for future research in more automated and safer spine surgery, some suggestions are given for potential research directions. More research should be performed into human tissue. Animal alternatives are easier to obtain both in the physical sense (e.g., from a butcher) as in the administrative sense (e.g., ethical approval) but there are still important anatomical and biomechanical differences between animal spines and human spines that limit the ability to thoroughly validate the sensor setup.

Sensor fusion was very limitedly present in this literature review. Only three studies investigated the effect of sensor fusion on the accuracy of the sensing method. All three studies showed improved outcomes when data from different sensors were fused. Preferably, the sensors that are fused should be of different sensing categories (e.g., from the force and torque category, from the sound and vibration category and from the bioelectrical properties category) as it is more likely to measure something with one sensor that a sensor from another category missed. The potential for sensor redundancy is much higher in that case.

This review also showed that the application of machine learning to decision making is very present in the case of robotic setups but almost completely lacking in non-robotic setups. It appears that there is a big research gap in machine learning applications for hand-held instruments in spine surgery. It is, nevertheless, an important direction as it combines the potential of multisensor technology and artificial intelligence with well-trained human senses and the experience of the surgeon. It also provides a larger sense of control for the surgeon than robotic setups do.

Finally, the next steps in sensing methods for spine surgery is to include more realistic situations such as a ‘breathing’ sample, which was shown to complicate drilling state identification by Jiang et al. [23]. Multisensor technology can be used to deal with this problem as part of the sensing modalities can be dedicated to monitor the motion of the sample, and as such, update the model in charge of state identification.

## 5. Conclusions

All sensors mentioned in this literature review, except for the EMG electrodes, show promising results in (robotic) spine surgery to improve the outcome of surgical procedures such as pedicle screw placement, laminectomy, discectomy, etc. Application in a robotic system is common for 6DOF force/torque sensors, microphones, and AE-sensors. Various studies provide a good example of how to integrate the sensor in the tool or robot seamlessly. In many cases, the simple thresholding of the measured signal is able to distinguish between operating a tool in cancellous or in cortical bone. Classifiers and neural networks can also perform real-time tissue classification and can accurately predict critical events during surgery such as breakthrough into soft tissue, given that the algorithm is trained well. The accuracy of the measurement systems discussed in this review was good, with many detection rates well over 90% when differentiation between different types of soft tissue is not taken into account. Redundancy by combining several different sensors and different measured features can improve this accuracy but synchronous data acquisition for all the sensors is essential. A research gap was found in the application of machine learning for data processing and decision making in the case of hand-held, non-robotic setups.

## Data Availability

Not applicable.

## Abbreviations

The following abbreviations are used in this manuscript:

AE	Acoustic Emissions
ANN	Artificial Neural Network
CNN	Convolutional Neural Network
CT	Computed Tomography
DOF	Degrees of Freedom
EMG	Electromyography
LCR	Inductance–Capacitance–Resistance
LSTM	Long Short-Term Memory
MISS	Minimally Invasive Spine Surgery
NPV	Negative Predictive Value
PSP	Pedicle Screw Placement
PPV	Positive Predictive Value
PLL	Posterior Longitudinal Ligament
PRISMA	Preferred Reporting Items for Systematic Reviews and Meta-Analysis
PSO	Particle Swarm Optimisation
SNR	Signal-to-Noise Ratio
SOFM	Self-Organising Feature Map
SVM	Support Vector Machine
TCE MEP	Transcranial Electrical Motor Evoked Potential
vBMD	Volumetric Bone Mineral Density
WPT	Wavelet Packet Transform
